# Inflammation of the Lateral Columns of the Pharynx Leading to Abscess Formation, with Report of Cases

**Published:** 1913-09

**Authors:** Henry L. Swain

**Affiliations:** New Haven


### Inflammation of the Lateral Columns of the Pharynx Leading to Abscess Formation, With Report of Cases.

Any isolated mass of lymphoid tissue can in a general way be expected to act when inflamed exactly after the fashion of the faucial tonsil, and can have, like the latter, simple, acute, follicular, rheumatic, diphtheritic and phlegmonous inflammations. The adenoid or pharyngeal tonsil may have acute as well as chronic inflammation. The same is true of the lingual tonsil. If we continue to remove root and branch from young children all of their adenoids and faucial tonsils, there will be abundant need to devote more and more attention to these masses of lymphoid tissue as well as to the lingual tonsil. In years gone by, when adenoids were removed from children and the faucial tonsils were left untouched, the latter subsequently enlarged into a perfectly healthy growth, as though needed by the system. If the faucials are thus ruthlessly removed, there will be enlargement of the lingual tonsil or lateral columns of the pharynx, as there has been of the faucials following the older simple adenoid operation. If this is logically true, we will find not only acute inflammations of these structures, but also phlegmons. We have assumed more or less arbitrarily that as the lateral column of the pharynx is no mean mass of lymphoid tissue, and as it has been known to have all other kinds of inflammation to which lymphoid tissue is heir, we have a logical right to expect that there may also be the phlegmonous type, and I propose to submit facts concerning certain cases confirmatory of this statement. I recently had six cases of edema of the larynx, in two of which there was no quinsy at all. There was inflammation of the lateral column of the cord in both these instances, and there was in one a marked general, what may be called rheumatic infection, where various joints of the body were affected, but with no persistence of the symptoms in any one place for any length of time. In these various cases of edema one has to look to some other cause than pressure.—Dr. Henry L. Swain, New Haven:

